# Extortion

**Published:** 1860-11

**Authors:** 


### EXTORTION.

(From *ex*, out of, and *torqueo*, to twist.)

THIS term, appropriately applied, has no reference to reward or equity of compensation, in exchange of commodities in common use. But little difficulty will present itself in the exchange of all things of determinate value; and hence, a standard, that commends itself to the consciousness, is apparent to all, so that there is no foundation for differences.

But in professions, where things above commercial, or mere money value are dealt in, the standard is a sliding scale, almost as various as the individuals with whom we come in contact, so that the matter becomes very complicated, and opens a door for endless controversy, if we admit false or doubtful elements or agents into the practice of the high callings denominated professions.

There is no mistake more fatal to the excellency of professional service, than to bring it to the standard of mere exchange or trade; for the plain reason that something, so etherial in its essence as to have eluded thus far the capabilities of lexicographers to fitly define, is the basis and utility of all pure professional function, that it doth not appear, as yet, how we can, consistently, drag it down to be measured by dollars and cents, or, as we might fitly say, abstract non-vital value.

This seems to withdraw the curtain a little, and to let a pencil of light penetrate our darkness, foreshadowing what is to be, viz: the exercise of all professions, as far as monetary interests are concerned, without compensation. Although to say there were no balance in such exercise of purely divine life forces, would pay but a poor compliment to the perception that could not see that it is more blessed to give than to receive; for He who gave more than any other has to give, first announced this beautiful statement.

What will a man *not* give in exchange for his life? No wonder that those earnest, honest, and persistent members of professions, who have attained eminent usefulness, command and obtain what the mere groundling deems extortionate compensation! It would really be extortionate, for the grovelling service such capabilities could, in their present imprisoned state, render; and, as honest men, they must enunciate the real sentiments of their inmost, for the service is *above* their appreciation, because it is not capable of measurement by non-vital value—a plane from which they have not yet emerged.

Convince men of their highest interests, and we shall never hear of extortion being applied to anything within their mightiest effort. No legitimate acquisition of real good can be regulated by or expressed in conventional value; or there would come a time that an unvarying market value would attach to each good, or means of good, and thus put an end to change, that is, progress.

Wherever skill is brought to bear upon any acquirement, the only true method of computing it is to count up the value of all that is in the direct line of its sequences; and you have a measure that all will acknowledge as adequate.

To save the life of an individual from impending loss, suffering, or death, is, to him, worth all things, and more; but it may be of very little consequence to the world at large.

Skill is the professional man's currency, so to speak, at this stage of the development of society; and no one would, for a moment, require him to exchange work for work, by the hour, day, month, or year, with the ditcher, and thus offset one against the other. And why? Let him who knows how, answer this query to his own consciousness; and he will very soon perceive that we are saddled with burdens of which we do not know the significance, until, after long and laborious experience, we have learned to attach superior value to superior effort, and superior mental power,—both of which have to lie behind all superior skill, and give it a charter to claim and receive the adequate return of its exercise.

Here, as elsewhere, the world is not slow to discern between the true and the seeming; for mere enormity of price does not always stand in relation with superiority of execution. All that is seeming is but one of the phases of gambling; while the true is the only truly professional prerogative.

Any return in the shape of remuneration to the professional man, is always, in the appreciation of the miser, *extortion*, really; for it does twist out of his grasp, per force of circumstances, all he ever devotes to the high uses of skill employed for his use or relief. It matters not as to the amount; for he will stand higgling for $\frac{1}{8}$ of one per cent. on a small sale or purchase with as much earnestness as he will demur to the payment of large amounts. With such minds, it matters not how cheap they obtain anything, they are miserable beyond the capability of liberal minds to be miserable about money, upon the discovery that they *might* have pressed down a per cent. lower, had they persisted a little longer.

Let the professional man be careful to obtain compatibility in all the relations of life. K.

